# Determinantes sociales de la salud que influyen en la prueba de Papanicolaou y mamografía en las mujeres chilenas

**DOI:** 10.15446/rsap.V27n3.114162

**Published:** 2025-05-01

**Authors:** Marco T. Bustos-Gutiérrez, Marjorie Liz Morales-Casetti, Olga A. Vásquez-Palma, Luis R. Bustos-Gutiérrez

**Affiliations:** 1 MB: Lic. Economía. Ph. D. Investigación en Ciencias Sociales. Académico, Universidad Católica de Temuco. Temuco, Chile. marco.bustos@uct.cl Universidad Católica de Temuco Universidad Católica de Temuco Temuco Chile marco.bustos@uct.cl; 2 MM: Ing. Civil Industrial. Ph. D. Investigación en Ciencias Sociales. Académica, Universidad de La Frontera, Departamento de Ingeniería Industrial y de Sistemas. Temuco, Chile. marjorie.morales@ufrontera.cl Universidad de La Frontera Universidad de La Frontera Departamento de Ingeniería Industrial y de Sistemas Temuco Chile marjorie.morales@ufrontera.cl; 3 OV: Antrop. Ph. D. Ciencias Sociales. Académica, Universidad Católica de Temuco. Temuco, Chile. ovasquez@uct.cl Universidad Católica de Temuco Universidad Católica de Temuco Temuco Chile ovasquez@uct.cl; 4 LB: MD. Alerg. Hospital Regional Militar de Mérida. Mérida, México. luisbustos9@gmail.com Hospital Regional Militar de Mérida Mérida México

**Keywords:** Papanicolaou, mamografía, cáncer, determinantes sociales de la salud, Chile *(fuente: DeCS, BIREME)*, Papanicolaou, mammography, cancer, social determinants of health, Chile *(source: MeSH, NLM)*

## Abstract

**Introducción:**

Se estima que en Chile el cáncer será la principal causa de mortalidad en los siguientes años. El Papanicolaou y la mamografía son los exámenes recomendados para detectar de manera precoz cáncer cervicouterino y de mama. La presencia de determinantes sociales de la salud negativos puede influir en la no toma de Papanicolaou y la mamografía en la población femenina chilena.

**Objetivo:**

Estimar la influencia que tienen los determinantes sociales de la salud en la no toma de exámenes de Papanicolaou y mamografía en mujeres chilenas.

**Método:**

Estudio descriptivo-analítico, transversal de base secundaria. Considera a la población femenina respondiente en la Encuesta de Caracterización Socioeconómica Nacional 2022, mayor de 18 años. Para mamografía N=19 220 mujeres. Para Papanicolaou N=33 992. Se realizaron análisis bivariados y multivariados de los determinantes sociales de la salud que pueden influir en la no toma de Papanicolaou y mamografía.

**Resultados:**

El no hacerse mamografía se asocia a estar desocupada, ser pobre, tener sistema de salud público, no haberse realizado un control de salud en más de 12 meses y no haberse realizado el Papanicolaou en los últimos tres años. Factores como edad, no hacerse un control de salud en más de 12 meses y no haberse realizado la mamografía en los últimos tres años favorecen la no toma de Papanicolaou.

**Conclusiones:**

Las mujeres chilenas con peores determinantes sociales de la salud son más propensas a la no realización de Papanicolaou y mamografía.

## Abstract (en)

**Introduction:**

In Chile cancer will be the main cause of mortality in the following years. Pap smears and Mammography are the recommended requirements to detect cervical and breast cancer early. The presence of negative social determinants of health can influence the non-taking of Pap smears and Mammography in the Chilean female population.

**Objective:**

Estimate the influence that social determinants of health have on not taking Pap smears and Mammography exams in Chilean women.

**Methods:**

Descriptive-analytical, cross-sectional secondary-based study. Considers the female population responding to the National Socioeconomic Characterization Survey 2022, over 18 years of age. For Mammography 19,220 women. For Pap smears 33,992 women. Bivariate and multivariate analyzes were carried out on the social determinants of health that may influence not taking Pap smears and Mammography.

**Results:**

Not having a Mammogram is associated with being unemployed, being poor, having a public health system, not having had a health check in more than 12 months and not having had a Pap smear in the last three years. Age, not having a health check-up in more than 12 months and not having had a Mammogram in the last three years, is associated with not having a Pap smear.

**Conclusions:**

Chilean women with worse social determinants of health are more likely to not have Pap smears and mammography.

El cáncer es el conjunto de enfermedades que tienen origen el crecimiento anómalo de células de los órganos o tejidos, condición que puede propagarse a otros órganos o tejidos del cuerpo 1 De acuerdo a The Global Cancer Observatory (GCO), el cáncer de mama (C50), pulmón (C33-34), colorrectal (C18-21), cuello uterino (C53) y tiroides (C73) son los tipos de cáncer más comunes en mujeres en todo el mundo 2-4. En 2022, en cuanto a la incidencia y la mortalidad, las tasas de C50 fueron 47,8 y 13,6, respectivamente, por cada 100 000 habitantes, mientras que para C53, la incidencia y la tasa de mortalidad fueron de 14,1 y 7,1, respectivamente por cada 100 000 habitantes 2. En 2019, el cáncer se posicionó como la principal causa de mortalidad en Chile 5.

Según GCO 6, se registraron 27 076 nuevos casos de cáncer diagnosticados en Chile en población femenina en 2022 -un aumento del 6,4% respecto a 2020-, de los cuales el 20,8% correspondió a C50 y el 5,8% a C53. La incidencia de C50 fue de 38,5 y la tasa de mortalidad fue de 10,3 por cada 100 000 habitantes. Para C53, la tasa de incidencia fue 11,3 y la tasa de mortalidad de 5,2 por cada 100 000 habitantes.

Luego de considerar que el cáncer será la principal causa de muerte en Chile hacia finales de la tercera década del presente milenio, se desarrolló el Plan Nacional contra el Cáncer 2018-2028 7. Este plan proporciona los servicios de atención de salud como su segunda línea de acción, que consiste en la provisión de servicios asistenciales. Esta línea busca mejorar la oferta de servicios de salud, indicando los procedimientos por seguir para tratamiento, cuidados paliativos y rehabilitación oncológica, además de hacer un fuerte énfasis en la detección precoz y el diagnóstico oportuno de C50 y C53 7.

Los exámenes de detección temprana y diagnóstico propuestos para C50 son la mamografía y el examen físico, teniendo como grupo objetivo a las mujeres entre 50 y 69 años. Para C53, el examen es el Papanicolaou en mujeres entre los 25 y 64 años 7,8. Es fundamental observar la presencia de cáncer en estos grupos etarios: en 2022, las mujeres chilenas en el grupo de edad de 50 a 69 años tuvieron una incidencia de 115,0 y mortalidad de 31,4 en C50. Las mujeres en el grupo de edad de 25 a 64 años tuvieron una prevalencia y mortalidad en C53 de 19,3 y 7,6, respectivamente 9.

La evidencia muestra que la detección temprana y el diagnóstico oportuno están asociados a una buena administración de tratamientos, incremento de la sobrevivencia y reducción de los costos de la atención 10. En el contexto latinoamericano, la oferta limitada de atención médica, el número limitado de pruebas de diagnóstico para los diferentes tipos de cáncer, la falta de personal técnico de laboratorio especializado y el escaso seguimiento de los resultados de los exámenes conllevan una baja detección temprana y tratamientos tardíos 1,11.

Según el Plan Nacional contra el Cáncer 2018-2028, aspectos negativos antes mencionados no deberían afectar a las mujeres en Chile. Sin embargo, la población femenina chilena presenta baja adherencia a la realización de exámenes para detectar de forma temprana C50 y C53 12-15.

Una explicación a esta situación podría residir en los determinantes sociales de la salud (DSS) de la población chilena. Los DSS se refieren al conjunto de factores no médicos que pueden influir en el estado de salud de la población 16,17^).^ Hacen referencia a dos dimensiones: la estructural y la intermedia. La primera, considera aspectos como la política macroeconómica, políticas y programas públicos vigentes, aspectos culturales y valores sociales que influyen sobre la posición social, el género, la etnicidad, el acceso a la educación y el empleo. La segunda dimensión, son factores que se distribuyen de acuerdo con la estratificación social, generando marcadas diferencias en exposición y vulnerabilidad 16,17. A esta segunda dimensión se asocian las circunstancias materiales, psicosociales, conductuales, biológicas, así como lo referente al sistema de salud 18,19. El acceso a la atención médica, la realización de exámenes diagnósticos, la distribución de los recursos sanitarios, entre otros aspectos, son influidos por los DSS de la población. En el caso de las mujeres, los DSS que pueden limitar la realización de Papanicolaou y mamografía 10-16,20-27, son el desconocimiento o la desconfianza respecto a los exámenes clínicos; largas listas de espera en el sistema de salud; dependencia de los sistemas públicos de atención de salud; bajo apoyo familiar, dependencia económica o dominación de la pareja o conviviente; ser indígena; vivir en condiciones de pobreza, zonas rurales o zonas alejadas de los centros de salud; tener un bajo nivel educacional; estar a cargo del cuidado de familiares; baja frecuencia de la asistencia a controles médicos; entre otros. Considerando lo anterior, este trabajo tiene como objetivo identificar los determinantes sociales de la salud asociados a la no realización de exámenes de Papanicolaou y mamografía en mujeres chilenas. Se hipotetiza que las mujeres con DSS desfavorables en el contexto chileno son menos propensas a realizarse dichos exámenes.

## MÉTODOS

### Diseño y participantes

Es un estudio descriptivo-analítico transversal de base secundaria. Se considera a la población femenina participante en la Encuesta de Caracterización Socioeconómica Nacional (CASEN) 2022 28. CASEN es una encuesta de representación nacional, regional y de zonas urbanas y rurales, diseño probabilístico, estratificado y bietápico, donde la unidad final de selección es la vivienda. Tiene un error muestral a nivel nacional absoluto de 0,3% y 4,3% de error relativo. El levantamiento de información de esta encuesta se llevó a cabo entre el 1 de noviembre de 2022 y el 2 de febrero de 2023. La población estudiada fueron las mujeres respondientes consideradas como informantes (respondieron cara-cara) con edad de 18 años y más.

### Variables

La adherencia a la prueba de Papanicolaou se evaluó preguntando: ¿en los últimos 3 años se ha hecho el Papanicolaou? Para medir la adhesión a la mamografía, se consideró la pregunta: ¿en los últimos 3 años se ha hecho una mamografía? En ambos casos se recodificó: 1=No, 0=Sí. Las variables analizadas como DSS estructurales fueron: condición de ocupación, escolaridad, etnia y ruralidad. Las variables analizadas como DSS intermedios fueron: edad, tener hijos, jefatura de hogar, estado civil, ser pobre, no tener cerca un centro de salud, tipo de sistema de salud, no haberse realizado un chequeo de salud en los últimos 12 meses. En la Tabla 1 se presenta un resumen de los DSS de este estudio.

**Tabla 1:** Resumen de los DSS del estudio

Edad Papanicolau	25-64 años
Edad mamografía	50-69 años
Escolaridad	0-27 (años de escolaridad)
Etnia	1=Sí (pertenece o se reconoce como perteneciente a un pueblo originario)
	0=No
Ruralidad	1=Rural
	0=Urbano
Tener hijos	1=Sí (uno o más)
	0=No
Jefa de hogar	1=Sí (se asume como jefe de hogar)
	0=No
Estado civil	1= Estar casada o tener pareja
	0=Soltera, divorciada, viuda, anulada
Condición de ocupación	1=Desocupada (desempleada/jubilada)
	0=Ocupada
Ser pobre	1=Ser pobre (estar por debajo de línea de pobreza)
	0=No ser pobre
Centro de salud cercano	1=No (centro de salud a más de 2,5 km)
	0=Sí
Tipo de sistema de salud	1=Público
	0=Privado
No haberse realizado un control de salud en los últimos 12 meses	1=No (el último control de salud fue hace más de 12 meses)
	0=Sí

### Análisis de los datos

En primer lugar, se calculó un resumen estadístico sobre la toma de exámenes Papanicolaou y mamografía. En segundo lugar, para evaluar la asociación en la no toma de mamografía y Papanicolaou, se realizaron modelos de regresión logística, analizando los resultados como Odds Ratio (OR), con intervalos de confianza del 95% (IC 95%). Las variables usadas como DSS estructurales e intermedios se consideraron de forma negativa o desfavorable 9. Se realizaron modelos de regresión logística bivariada y multivariada para observar la relación entre los DSS y la no toma de mamografía y Papanicolaou. En el modelo multivariado se consideraron los DSS que fueron significativos en el análisis bivariado. Para todos los análisis se utilizó el software SPSS v.23. El Ministerio de Salud de Chile 8 sugiere que para la prueba de Papanicolaou las mujeres deben tener entre 25 y 64 años, y para mamografía, entre 50 y 69 años. Aspectos que se tuvieron en cuenta al momento de seleccionar los datos de las mujeres participantes de la encuesta CASEN.

### Consideraciones éticas

La participación en la encuesta CASEN 2022 18 es voluntaria y anónima. Como es una base de datos pública, accesible bajo la supervisión del Ministerio de Desarrollo Social y Familia de Chile (MINSAL), no es necesario que la investigación sea revisada previamente por un comité de ética.

## RESULTADOS

Las mujeres dentro del grupo de edad elegible para la prueba de Papanicolaou tenían una edad promedio de 45,6 años y 12 años de formación escolar, respectivamente. La edad promedio y los años de formación escolar para las mujeres dentro del grupo etario que puede realizarse mamografía fue de 59 años y 10 años, respectivamente. En el caso de las mujeres chilenas consideradas en este estudio, el 27,0% y el 32,3% no se habían realizado examen de Papanicolaou y mamografía, respectivamente, en los tres años previos a la realización de la encuesta. Cuando se consulta por los motivos para no realizarlos, la causa principal es el poco interés sobre los exámenes de Papanicolaou y mamografía. Le siguen los motivos: el poco tiempo que tienen para destinar a este tipo de exámenes y las barreras institucionales (horarios de consulta o dificultad para agendar citas médicas). Estas tres razones representan el 65,5% de la baja adhesión en el caso de mamografía y el 67,5% en el caso del Papanicolaou. El aspecto monetario es una condición que afecta relativamente poco la toma de Papanicolaou y mamografía, a diferencia del temor o la incomodidad de las pruebas y la creencia que no les corresponde realizarlas (Tabla 2).

**Tabla 2:** Adhesión a Papanicolaou y mamografía y razones de no toma de exámenes de mujeres chilenas, 2022

Realizaron examen	Mamografía (n=19 220) (%)	Papanicolaou (n=33 992) (%)
Sí	67,20	72,50
No	32,30	27,00
No sabe/no recuerda	0,50	0,50
Razones de no toma de exámenes	Mamografía	Papanicolaou
Desconocimiento	4,20	4,50
Temor/incomodidad	11,00	8,80
Poca importancia/olvida hacerlo	36,00	38,00
Institucionales	15,80	11,20
Tiempo	13,80	18,40
Falta de dinero	2,60	1,70
No le corresponde	7,80	9,40
Otra	9,00	8,00

Nota: De acuerdo al Ministerio de Salud 7, para Papanicolaou se considera a la población entre 25 y 64 años de edad, para mamografía a la población entre 50 y 69 años de edad. Fuente: Elaboración propia con datos de CASEN 2022 18.

En el análisis bivariado, la proximidad a un centro de salud cercano y ser indígena no fueron estadísticamente significativos para el caso de no realizarse mamografía. En el caso de no realizarse la prueba de Papanicolaou, no fueron significativas ser jefa de hogar, no tener centro de salud cercano y el tipo de sistema de salud que poseen (Tabla 3).

**Tabla 3:** Análisis bivariado y regresión logística múltiple de la no toma de Papanicolaou y mamografía en mujeres chilenas mayores de 18 años

	Mamografía		Papanicolaou	
OR (IC 95%)	OR Ajustado (IC 95%)	OR (IC 95%)	OR Ajustado (IC 95%)
Edad	1,035	0,988	1,01	1,085
	(1,029-1,041)	(0,980-0,995)	(1,008-1,012)	(1,080-1,090)
Escolaridad	0,972	0,965	0,988	1,006
	(0,964-0,979)	(0,954-0,975)	(0,983-0,993)	(0,996-1,016)
Etnia	1,064		0,917	0,836
	(0,969-1,169)		(0,855-0,983)	(0,758-0,921)
Rural	0,854	0,872	0,819	0,847
	(0,791-0,922)	(0,788-0,965)	(0,769-0,872)	(0,776-0,925)
Tener hijos	0,6	0,868	0,616	0,642
	(0,533-0,675)	(0,741-1,017)	(0,574-0,661)	(0,569-0,724)
Jefa de hogar	1,132	1,03	0,99	
	(1,060-1,209)	(0,932-1,138)	(0,942-1,041)	
Estado civil	0,751	0,852	0,81	0,926
	(0,705-0,799)	(0,774-0,938)	(0,772-0,850)	(0,864-0,991)
Desocupada	1,184	1,11	1,148	1,071
	(1,111-1,263)	(1,015-1,214)	(1,094-1,205)	(0,997-1,150)
Pobre	1,205	1,216	1,136	1,065
	(1,065-1,363)	(1,033-1,432)	(1,047-1,232)	(0,949-1,195)
Cercanía centro de salud	1,01		0,974	
	(9,39-1,0879		(0,920-1,032)	
Tipo sistema de salud	1,314	1,39	1,053	
	(1,168-1,479)	(1,191-1,624)	(0,975-1,138)	
Control de salud	2,7	2,085	2,623	2,127
	(2,528-2,884)	(1,916-2,269)	(2,497-2,756)	(1,990-2,274)
Papanicolaou	19,26	18,423 (16,955-20,018)	---	---
	(17,804-20,836)			
Mamografía	---	---	10,35	15,069
			(9,703-11,039)	(13,973-16,252)
Logaritmo de la verosimilitud -2		15 352,396		22 473,895
R2 Cox y Snell		0,331		0,268
R2 Nagelkerke		0,462		0,39
Porcentaje global clasificación		82,6		81,8
Constante		0,215		0,002

Nota: Los valores en negrita fueron significativos al 95%.

En el modelo ajustado los DSS que se asocian a la toma de mamografía son la edad (OR: 0,988), los años de escolaridad (OR: 0,965), vivir en un contexto rural (OR:0,872), estar casada o contar con pareja (OR: 0,852). El no hacerse mamografía se asocia a estar desocupada (OR:1,110), ser pobre (1,216), tener sistema de salud público (1,390), no haberse realizado un control de salud en más de 12 meses (2,085) y no haberse realizado el Papanicolaou (OR:18,423). Por otro lado, pertenecer a un pueblo originario (OR: 0,836), tener hijos (OR:0,642) y estar casada (OR: 0,926) fungen como DSS que favorecen la toma de Papanicolaou. La edad (OR: 1,085), no hacerse un control de salud en más de 12 meses (OR:2,127) y no haberse realizado la mamografía (OR: 15,069) influyen la no toma de Papanicolaou.

## DISCUSIÓN

La Organización Mundial de la Salud 1 ha advertido sobre el incremento de cáncer en sus diversas formas en todos los países, así como los efectos nocivos de su carga (física, emocional y financiera), tanto para los sistemas de salud como para los enfermos y sus respectivas familias. Los factores genéticos y los factores de riesgo 7 presentes en la población pueden verse influidos de forma negativa por los DSS, complejizando la detección y el tratamiento del cáncer. Se ha identificado a los DSS como factores que generan una gran diferencia en el diagnóstico temprano, la aplicación de tratamiento y la posterior supervivencia de los pacientes con cáncer, sobre todo en aquellos países donde hay alta vulnerabilidad económica y con sistemas de salud débiles 11,12,19. En Chile, el C50 y el C53 se encuentran entre los cinco tipos de cáncer de mayor incidencia en la población femenina 6,7,13,15,29, en un contexto en el que C50 se perfila como la principal causa de mortalidad en este segmento de la población 5.

En Chile, las políticas y los programas públicos (DSS estructurales) buscan la detección temprana de C50 y C53 7,15,30. Aun así, deben mejorar su incidencia en la población femenina, ya que muchas mujeres no se someten Papanicolaou y mamografía (Tabla 1). Entre las razones que dieron las chilenas para no tener exámenes diagnósticos destacan: falta de importancia u olvido, falta de tiempo y factores institucionales (horario de consultas o dificultad para programar exámenes). Estos aspectos pueden ser considerado DSS intermedios. Las razones antes mencionadas coinciden con estudios previos que buscaron determinar la baja adherencia a la toma de Papanicolaou y mamografía para la población femenina chilena 12,13,15,25,26.

Los DSS que favorecen realizarse examen de Papanicolaou son pertenecer o reconocerse como parte de un pueblo originario, vivir en contexto rural, tener hijos y estar casada o contar con pareja. Los factores que influyen en la no realización de examen de Papanicolaou son la edad, no haberse realizados un control o chequeo médico en más de 12 meses y no haberse realizado mamografía en los últimos tres años. Los DSS estructurales estar desocupada y ser pobre se consideran determinantes que influyen en la no toma de Papanicolaou, pero en el análisis multivariado no fueron significativos.

Los DSS estructurales que favorecen la toma de mamo-grafía fueron la escolaridad y vivir en un contexto rural. Como DSS estructurales que afectan de forma negativa la toma de mamografía se encontraron el estar desocupada y ser pobre. Los DSS intermedios que afectan negativamente fueron el tener sistema público de salud, no realizarse un control o chequeo médico en más de 12 meses y no haberse realizado Papanicolaou en los últimos tres años.

Los resultados de los modelos bivariado y multivariado respecto de los DSS que influyen en la toma de Papanicolaou y mamografía son similares a otros realizados en mujeres chilenas 13,15,25,26, españolas 23 e indonesias 24. En esta investigación se consideró la variable residir en zona urbana como variable de referencia, buscando observar la presencia del efecto negativo que tiene sobre la realización de exámenes diagnóstico el vivir en contexto rural, pero los resultados obtenidos muestran un efecto contrario al esperado.

Encontramos que la posición económica y el tipo de sistema de salud que tienen las mujeres son DSS que influyen en la realización de exámenes de Papanicolaou y mamografía. De igual manera, se observa el efecto negativo que tienen sobre la toma de Papanicolaou y mamo-grafía los horarios de las consultas o de exámenes; esto puede ser explicado por dos motivos: la saturación, pues el sistema público chileno atiende al 77% de la población 31, y la rigidez del mundo del trabajo, donde no hay permisos específicos para ausentarse y acudir a la realización de exámenes de Papanicolaou y mamografía, por lo que las mujeres deben buscar horarios que no afecten sus actividades del hogar o laborales. Una diferencia del sistema de salud chileno con otros contextos, es que el tratamiento de cáncer se encuentra cubierto por el programa de Garantías Explícitas en Salud (GES), cobertura que cubre la atención de C50 y C53. Sin embargo, por lo antes expuesto, esta cobertura de salud resulta más reactiva que preventiva, afectando en consecuencia la salud de las mujeres.

Entre las limitaciones del estudio podemos mencionar las siguientes: en primer lugar, es un estudio transversal, que no nos permite observar causalidad entre exposición y efectos, y además se limita al número de participantes en la encuesta utilizada. En segundo lugar, en Chile las mujeres tienen acceso a servicios públicos o privados de salud, incluidos los migrantes no regulares, que pueden acudir al sistema de salud público; por lo tanto, este estudio no proporciona evidencia de mujeres sin cobertura médica. En tercer lugar, el estudio deja de lado la influencia de aspectos culturales (religión, por ejemplo) o acceso a infraestructuras públicas. Cuarto, no observamos si la discapacidad en las mujeres genera diferencias en los exámenes de Papanicolaou y mamografía. Estas limitaciones pueden convertirse en investigaciones futuras.

Las mujeres en Chile con DSS desfavorables tienen menos probabilidades de realizarse pruebas de Papanicolaou y mamografía que mujeres chilenas con DSS más favorables. Los resultados de esta investigación pueden utilizarse para actualizar la objetivos o acciones del Plan Nacional contra el Cáncer 2018-2028 6 y promover la educación y la importancia de la realización de Papanicolaou y mamografía en mujeres chilenas. Dado el nivel de asociación entre Papanicolaou y mamografía, es necesario reforzar las instancias que promuevan el desempeño de estas pruebas y la realización de controles médicos con mayor frecuencia. Tomando en consideración que mayoritariamente la cobertura de salud es provista por el sector público, los cambios deben pasar primero el filtro de los tomadores de decisión antes de poder ser implementadas por el sistema de salud, es decir, modificar los DSS estructurales e intermedios existentes.
